# Positive Coping as a Mediator of Mobile Health Intervention Effects on Quality of Life Among People Living With HIV: Secondary Analysis of the Randomized Controlled Trial Run4Love

**DOI:** 10.2196/25948

**Published:** 2022-02-17

**Authors:** Yu Zeng, Yan Guo, Rainbow Tin Hung Ho, Mengting Zhu, Chengbo Zeng, Aliza Monroe-Wise, Yiran Li, Jiaying Qiao, Hanxi Zhang, Weiping Cai, Linghua Li, Cong Liu

**Affiliations:** 1 Department of Medical Statistic School of Public Health Sun Yat-sen University Guangzhou China; 2 Longgang Center for Disease Control and Prevention in Shenzhen Shenzhen China; 3 Department of Population and Quantitative Health Sciences University of Massachusetts Chan Medical School Worcester, MA United States; 4 Department of Social Work & Social Administration The University of Hong Kong Hong Kong China; 5 Centre on Behavioral Health The University of Hong Kong Hong Kong China; 6 The Jockey Club School of Public Health and Primary Care Faculty of Medicine The Chinese University of Hong Kong Hong Kong China; 7 Department of Global Health and Social Medicine Harvard Medical School Boston, MA United States; 8 Department of Global Health University of Washington Seattle, WA United States; 9 Shanghai Center for Disease Control and Prevention Shanghai China; 10 National Center of AIDS/Sexually Transmitted Disease Control and Prevention Chinese Center for Disease Control and Prevention Beijing China; 11 Department of Infectious Diseases Guangzhou Number Eight People’s Hospital Guangdong China

**Keywords:** mediation effect, mobile health, quality of life, positive coping, HIV, randomized controlled trial

## Abstract

**Background:**

The effectiveness of psychosocial interventions on quality of life (QOL) among people living with HIV has been validated, including mobile health (mHealth) interventions. However, it is unclear which components of such interventions account for these effects.

**Objective:**

This study aims to examine positive coping as a potential mediator of the effects of an mHealth intervention on QOL among people living with HIV.

**Methods:**

For this secondary analysis, we used data from an mHealth-based randomized controlled trial, Run4Love, which was conducted to improve QOL and mental health outcomes of people living with HIV. A total of 300 participants were randomly assigned to the intervention group to receive the adapted cognitive-behavioral stress management courses and regular physical activity promotion or the waitlist control group in a 1:1 ratio. Our analysis focused on positive coping and QOL, which were repeatedly measured at baseline and at 3-, 6-, and 9-month follow-ups. Latent growth curve models were constructed to explore the mediating role of positive coping in the effects of the mHealth intervention on QOL.

**Results:**

Positive coping served as a mediator in the effect of the mHealth intervention on QOL for up to 9 months. The mHealth intervention had a significant and positive indirect effect on the slope of QOL via the slope of positive coping (*b*=2.592×1.620=4.198, 95% CI 1.189-7.207, *P*=.006). The direct effect of the intervention was not significant (*b*=0.552, 95% CI −2.154 to 3.258, *P*=.69) when controlling for the mediator.

**Conclusions:**

The longitudinal findings suggest that positive coping could be a crucial mediator of the mHealth intervention in enhancing QOL among people living with HIV. These findings underscore the importance of improving positive coping skills in mHealth interventions to improve QOL among people living with HIV.

## Introduction

### Background

Substantial improvements to and increased coverage in antiretroviral therapy have resulted in extended life expectancy of people living with HIV, leading to over 1.25 million HIV seropositive survivors in China [1,2]. With improved survival, quality of life (QOL) during treatment has become increasingly important for people living with HIV [3]. Owing to HIV disease progression and treatment side effects, the QOL of people living with HIV continues to be lower than that of people living with other chronic diseases, as well as the general population [4,5]. Poor QOL is not only related to suboptimal adherence to antiretroviral therapy, leading to inferior therapeutic effects [6], but is also associated with elevated HIV risk behavior, leading to increased risk of HIV transmission in the community [7]. Therefore, effective interventions to improve QOL in people living with HIV are warranted, especially those that can reach a large number of the targeted population, such as mobile health (mHealth) programs.

The literature suggests that psychosocial interventions such as cognitive-behavioral stress management (CBSM) programs can improve QOL in a variety of populations with chronic diseases, including people living with HIV [8-12]. Evidence also shows that mHealth-based psychosocial interventions produce similar benefits in comparison with face-to-face interventions, and they have additional advantages because of the easy accessibility and cost-effectiveness [13,14]. Nevertheless, the potential mechanism by which these mHealth interventions improve QOL among people living with HIV remains unclear. There might be various factors that affect the outcomes of the intervention. Mediation constitutes a mechanism through which the independent variable affects the dependent variable of interest [15]. Exploration of mediators will help understand the effective components of an intervention, which is crucial for future optimization of the program design [16].

A considerable amount of research has focused on exploring factors associated with QOL, with positive coping being one factor that has received substantial attention [17-21]. Positive coping is defined as individuals’ active cognitive response (eg, trying to see the positive side) and adaptive behavioral response (eg, talking with family or friends) when managing stressful situations [22]. A substantial body of cross-sectional studies have shown that higher positive coping is related to higher QOL [18-20]. Some randomized controlled trials (RCTs) have indicated that positive coping might be a key determinant of the effectiveness of psychosocial interventions in improving QOL among people living with HIV [23,24]. For example, Hansen et al [24] found that HIV-positive participants reported significant postintervention improvement in both QOL and positive coping in the cognitive-behavioral support group, as well as in the control group providing mental health sessions and psychiatric services upon individual request. Using mixed models, the study also found that positive coping was directly associated with improved QOL in multiple time points across the intervention [24]. Similarly, another study revealed that a CBSM intervention was effective in improving positive coping in HIV-seropositive men who have sex with men, and positive coping might be an important predictor of QOL improvement [23].

Previous studies exploring the mediators of the effects of interventions on improving QOL have mostly been conducted in face-to-face settings [23,25,26]. It is unclear whether the factor mediating the effects of an intervention on QOL in face-to-face settings remains effective in mHealth settings. Furthermore, existing studies on mediation analysis mostly use pre- and postintervention data, whereas interventions in repeated measure design are lacking [11,23,25]. As 2–time point data contain limited information on individual changes, longitudinal data tracking over multiple time points (≥3) may allow further investigation on the changes of both the mediator and the outcome, as well as the trajectory of the time-varying relationships between them over time [27].

### Objective

To bridge the gaps in the existing literature, we used longitudinal data from the Run4Love study to examine the mediating role of positive coping on QOL in an mHealth intervention. The Run4Love study was a WeChat (Tencent)-based RCT to examine the intervention effects of an adapted CBSM course with physical activity promotion compared with usual care in people living with HIV. In this study, we hypothesized that the mHealth intervention would enhance the use of positive coping strategies in people living with HIV, which in turn was related to the significant improvement in QOL over time.

## Methods

### Research Setting and Ethical Considerations

The study used data from an mHealth-based RCT, Run4Love (ChiCTR-IPR-17012606) [28]. The Run4Love intervention was designed to improve mental health outcomes of people living with HIV, including, but not limited to, the reduction in depressive symptoms and improvement in QOL [28,29]. It was conducted from September 2017 to October 2018 in Guangzhou, the third-largest city of China [30]. A total of 300 participants were randomly assigned to either the intervention group to receive a 3-month WeChat-based mHealth program or the waitlist control group to receive usual care in a 1:1 ratio. The study design, recruitment procedures, and process of randomization were detailed in the study protocol, which was approved by the Institutional Review Board of Sun Yat-sen University [28].

### Participants Enrollment

The trained research staff recruited participants from the infectious disease outpatient department of the only hospital designated for HIV care and treatment in Guangzhou. Patients who showed interest in the study were invited to participate in a consultation session to receive further information about the program and complete a screening questionnaire. Patients were eligible to participate if they met all of the following inclusion criteria: (1) aged ≥18 years, (2) HIV seropositive, (3) having elevated depressive symptoms (Center for Epidemiologic Studies-Depression score ≥16), and (4) using WeChat, the most popular instant messaging mobile program in China, with more than 1 billion active users worldwide [31]. Patients were excluded if they met any of the following exclusion criteria: (1) currently on psychotherapy or taking psychiatric drugs, (2) unable to complete the questionnaires, (3) unable to read or listen to the intervention materials, and (4) unable to engage in physical activities for medical reasons.

Participants who met the aforementioned eligibility criteria and provided written informed consent were enrolled in the study. Under the guidance of the research staff, participants were asked to complete electronic questionnaires in the outpatient department at baseline and 3-, 6-, and 9-month follow-ups. Participants would receive 50 RMB (ie, approximately US $8) or gifts of equivalent value (eg, a yoga mat) as an incentive for completing each assessment.

### The Run4Love Intervention

Participants in the intervention group received a 3-month WeChat-based intervention, which consisted of the adapted CBSM course and physical activity promotion [28]. CBSM is a therapeutic approach to teaching people living with HIV to manage their stress by focusing on how individuals’ thoughts affect their emotions and behaviors and to influencing participants’ irrational thoughts and changing their thought patterns and behaviors [32]. The adapted CBSM course mainly included coping and stress reduction skills such as meditation, muscle relaxation, and abdominal respiration training. The physical activity promotion program mainly included guidance on exercise, benefits of exercise, and a healthy diet. The content of these 2 intervention components was provided through 65 intervention materials in multiple formats, including short articles, motivational posters, and audio clips. These materials were delivered to participants on a near-daily basis (5-6 times a week) via an enhanced WeChat platform, the Run4Love platform. On average, each of the short articles had around 1300 words, which required about 5 minutes to read; each poster with motivational messages required about half a minute to read; each audio recording required 5 to 10 minutes to listen to [33].

Participants in the control group received a brochure on nutrition and healthy living style. They would be offered the Run4Love program as soon as the study was completed (ie, 9 months after their enrollment).

### Measurement

#### QOL Assessment

QOL was measured using the 31-item World Health Organization Quality of Life HIV short version (WHOQOL-HIV BREF) at baseline and 3, 6, and 9 months. The WHOQOL-HIV BREF has been widely used among people living with HIV with proven reliability and validity [34,35]. The 31-item scale measures QOL across six domains (ie, physical, psychological, level of independence, social relationships, environment, and spirituality) over the last 2 weeks. Each item was rated on a 5-point Likert scale ranging from 1 (not at all) to 5 (extremely), altogether providing a 4-20 score in each domain and a 24-120 score for the whole scale after the transformation, with higher scores indicating better QOL [34]. An example of these items assessing QOL was “How much do you enjoy life?” Good reliability of the WHOQOL-HIV BREF was shown in the study, and Cronbach α ranged from .727 to .838 across the 4 assessment points.

#### Positive Coping

Positive coping was measured using the 12-item subscale of the Simplified Ways of Coping Questionnaire with good reliability and validity in the Chinese populations [36-38]. The subscale consists of 12 items such as “Try to look on the bright side of things” and “Find out several different methods to solve the problem” to assess the frequency of positive attitudes and skills of coping that a participant adopted in daily life. Each item was rated on a 4-point Likert scale ranging from 0 (not use at all) to 3 (use frequently), providing a 0-36 score, with higher scores indicating higher levels of positive coping. Cronbach α ranged from .848 to .925 across the 4 assessment points in this study.

#### Demographic Characteristics

Demographic characteristics included age, gender, marital status, educational level, sexual orientation, employment status, family monthly income, and duration of HIV infection (years).

### Statistics Analysis

The intention-to-treat principle was applied to all analyses in this study. Descriptive analyses of demographic characteristics, QOL, and positive coping were performed. Differences in outcome measures and baseline characteristics between the intervention and control groups were evaluated using the 2-tailed *t* test or Wilcoxon rank-sum test for continuous variables and the chi-square test for categorical variables. Latent growth curve models (LGCMs) were constructed to examine the mediating role of positive coping in the effects of the mHealth intervention on QOL among people living with HIV. All statistical hypothesis tests were 2-sided, and a *P* value <.05 was considered statistically significant. Descriptive analyses and group comparisons of the key variables were conducted using R (version 3.5), and LGCMs were constructed using Mplus (version 7; Muthén & Muthén). Missing data in all LGCMs were managed using the robust maximum likelihood estimation procedure.

The analyses of the mediation effect were conducted in 3 steps [39]. First, 2 unconditional LGCMs were constructed separately to estimate the growth trajectories of the outcome measure (QOL) and potential mediator variable (positive coping). The latent variables of intercept and slope (ie, the initial status and rate of change) were estimated from the repeated measures at 4 time points. For the intercept factor, the loadings across the 4 assessment points were fixed to 1. For the slope factor, the first loading was fixed to 0, the second to 1, and the other loadings were freely estimated [40]. Instead of assuming a linear trajectory by setting the loadings onto the slope factor to 0, 1, 2, and 3, it was more reasonable to explore the potentially nonlinear trajectories by setting the third and fourth loadings free when the assumption of linear LGCM was unconfirmed [40].

Second, 2 conditional LGCMs were specified to separately examine the impact of the intervention on QOL and positive coping. The conditional models were extensions of the unconditional models to incorporate the variable of intervention group assignment (ie, intervention group=1 and control group=0) as a covariate.

Third, a parallel-process LGCM was constructed to evaluate whether the intervention was effective in improving QOL via the mediator variable of positive coping. The parallel-process model was a combination of the aforementioned 2 conditional LGCMs, which simultaneously estimated the trajectories of QOL and positive coping, and incorporated intervention group assignment as a covariate. The mediation effect was tested based on bias-corrected 95% bootstrapped CIs with a resampling of 5000 [41,42].

Model fit was evaluated using chi-square test statistics and other indexes, including the Tucker-Lewis index (TLI), the comparative fit index (CFI), the root mean square error of approximation (RMSEA), and the standardized root mean square residual (SRMR). An LGCM with adequate model fit should meet the following criteria: TLI>0.90, CFI>0.90, RMSEA<0.08, and SRMR<0.08 [43-45].

## Results

### Baseline Characteristics

Descriptive statistics for baseline characteristics are shown in Table 1. The baseline characteristics of the participants were balanced between the intervention and control groups, except for the fact that a slightly higher proportion of heterosexual participants were allocated to the control group. The median age of the participants was 27.5 years, and the median duration of HIV infection was 1.7 years. The majority were male (277/300, 92.3%), well-educated (182/300, 60.7% with at least some college education), unmarried (262/300, 87.3%), and employed (251/300, 83.7%) and had a moderate level of income (176/300, 58.7% with family monthly income <7000 yuan [US $1100]). Up to 81.7% (245/300) of the participants were homosexual, bisexual, or uncertain of their sexuality.

**Table 1:** Baseline Characteristics of the participants in the Run4Love randomized controlled trial (N=300).

Variables		Total (N=300)	Intervention (n=150)	Control (n=150)	*P* value	
Age (years), median (IQR)		27.5 (24.5-31.3)	27.4 (24.3-31.1)	27.8 (24.6-32.2)	.29	
**Gender, n (%)**						.13
	Male	277 (92.3)	142 (94.7)	135 (90)		
	Female	23 (7.7)	8 (5.3)	15 (10)		
**Educational level, n (%)**						.10
	>High school	182 (60.7)	98 (65.3)	84 (65)		
	≤high school	118 (39.3)	52 (34.7)	66 (35)		
**Marital status, n (%)**						.73
	Single or divorced or widowed	262 (87.3)	132 (88)	130 (86.7)		
	Married	38 (12.7)	18 (12)	20 (85.3)		
**Employment status, n (%)**						.29
	Employed	251 (83.7)	123 (82)	128 (85.3)		
	Unemployed	49 (16.3)	27 (18)	22 (14.7)		
**Family monthly income (yuan), n (%)**						.20
	≥7000 (US $1100)	124 (41.3)	68 (45.3)	56 (37.3)		
	<7000 (US $1100)	176 (58.7)	82 (55.7)	94 (62.7)		
**Sexual orientation, n (%)**						.03
	Homosexual or bisexual or uncertain	245 (71.7)	130 (86.7)	115 (76.7)		
	Heterosexual	55 (18.3)	20 (13.3)	35 (23.3)		
Duration of HIV infection (years), median (IQR)		1.7 (0.6-3.7)	1.7 (0.6-4.0)	1.8 (0.6-3.9)	.62	
Positive coping, mean (SD)		18.4 (5.8)	18.4 (5.5)	18.3 (6.2)	.92	
Quality of life, mean (SD)		77.0 (9.2)	77.4 (9)	76.6 (9.4)	.44	

### Changes in Repeated Measures

**Figure 1:**
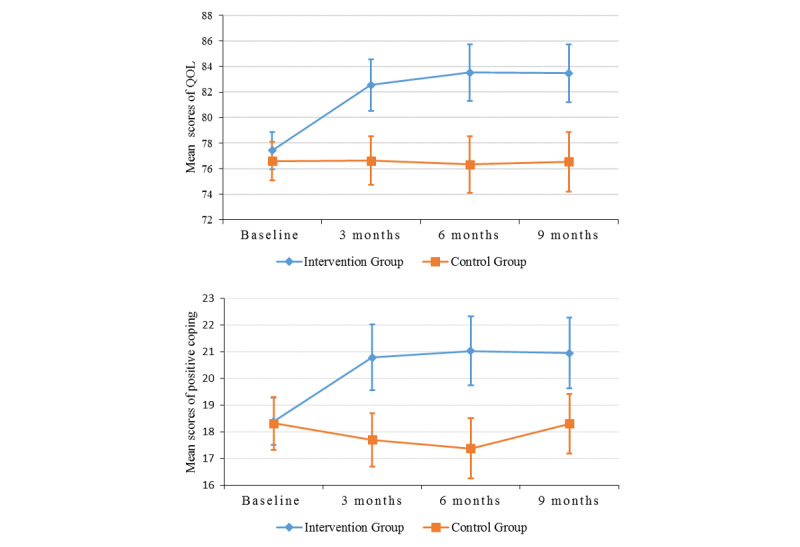
Repeated measures of quality of life (QOL) and positive coping of the participants in the intervention and control groups over time. Error bars represent 95% CIs.

**Figure 2:**
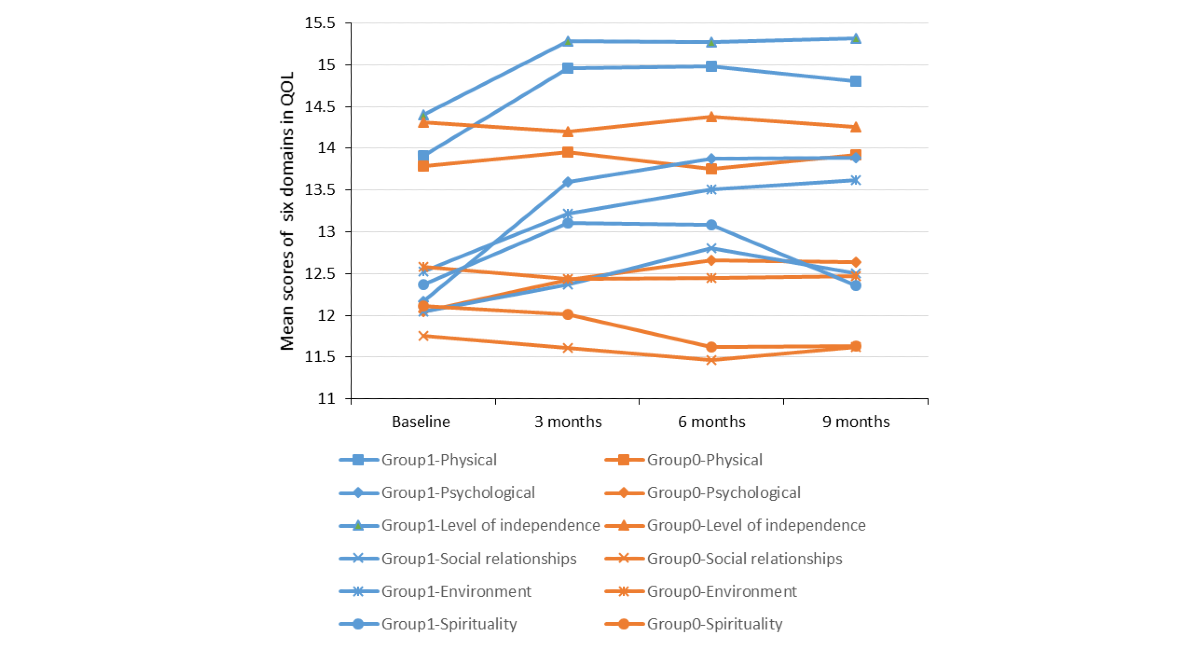
Repeated measures of the 6 domains in quality of life (QOL) of the participants in the intervention and control groups over time. Group 1 and Group 0 represent the intervention and control groups, respectively.

Repeated measures of the outcome variable (ie, QOL) and potential mediator (ie, positive coping) at the 4 assessment points are presented in Table 2. The sample mean and 95% CIs of QOL and positive coping over 9 months for both the intervention and control groups are presented in Figure 1. Participants in the intervention group had significantly higher levels of QOL and positive coping than those in the control group at the 3-, 6-, and 9-month follow-ups, indicating significant effects of the Run4Love mHealth intervention on improving QOL and positive coping over time. Specifically, in comparison with those in the control group, participants in the intervention group also had significantly higher mean scores in all 6 domains of QOL at the 3-, 6-, and 9-month follow-ups (Figure 2; Table 3).

**Table 2:** Repeated measures of quality of life and positive coping of the participants in the Run4Love randomized controlled trial.

Variables	Quality of life, mean (SD)			Positive coping, mean (SD)		
	Intervention^a^	Control^b^	*P* value	Intervention^a^	Control^b^	*P* value
Baseline	77.43 (9.03)	76.59 (9.42)	.44	18.39 (5.46)	18.32 (6.15)	.92
3 months	82.54 (12.03)	76.63 (11.08)	<.001	20.79 (7.33)	17.70 (5.88)	<.001
6 months	83.51 (12.88)	76.32 (12.96)	<.001	21.03 (7.48)	17.38 (6.59)	<.001
9 months	83.48 (13.17)	76.54 (13.34)	<.001	20.95 (7.75)	18.31 (6.41)	.003

^a^For variables in the intervention group, the sample sizes were 150, 139, 132, and 133 at baseline and 3-, 6-, and 9-month follow-ups, respectively.

^b^For variables in the control group, the sample sizes were 150, 135, 133, and 127 at baseline and 3-, 6-, and 9-month follow-ups, respectively.

**Table 3:** Repeated measures of the 6 domains in quality of life of the participants in the Run4Love randomized controlled trial.

Domains in quality of life		Intervention^a^	Control^b^		*P* value
**Physical**					
	Baseline	13.91 (2.05)	13.79 (2.39)	.66	
	3 months	14.96 (2.45)	13.95 (2.60)	.001	
	6 months	14.98 (2.69)	13.75 (2.77)	<.001	
	9 months	14.80 (2.84)	13.92 (3.00)	.02	
**Psychological**					
	Baseline	12.17 (2.17)	12.04 (2.08)	.60	
	3 months	13.60 (2.50)	12.42 (2.24)	<.001	
	6 months	13.88 (2.54)	12.66 (2.73)	<.001	
	9 months	13.89 (2.83)	12.64 (2.60)	<.001	
**Level of independence**					
	Baseline	14.40 (1.81)	14.31 (2.10)	.70	
	3 months	15.28 (2.04)	14.20 (2.27)	<.001	
	6 months	15.27 (2.23)	14.38 (2.26)	.002	
	9 months	15.32 (2.29)	14.26 (2.38)	<.001	
**Social relationships**					
	Baseline	12.05 (2.13)	11.75 (2.10)	.22	
	3 months	12.37 (2.70)	11.61 (2.22)	.01	
	6 months	12.80 (2.54)	11.46 (2.62)	<.001	
	9 months	12.50 (2.53)	11.62 (2.42)	.005	
**Environment**					
	Baseline	12.52 (1.93)	12.58 (2.08)	.80	
	3 months	13.22 (2.44)	12.44 (2.15)	.005	
	6 months	13.51 (2.45)	12.45 (2.74)	.001	
	9 months	13.62 (2.35)	12.47 (2.56)	<.001	
**Spirituality**					
	Baseline	12.37 (3.00)	12.11 (3.22)	.47	
	3 months	13.11 (3.16)	12.01 (3.29)	.005	
	6 months	13.08 (3.37)	11.62 (3.36)	<.001	
	9 months	13.36 (3.22)	11.63 (3.49)	<.001	

^a^For variables in the intervention group, the sample sizes were 150, 139, 132, and 133 at baseline and 3-, 6-, and 9-month follow-ups, respectively.

^b^For variables in the control group, the sample sizes were 150, 135, 133, and 127 at baseline and 3-, 6-, and 9-month follow-ups, respectively.

The dropout rates were 8.7% (26/300; 11/150, 7.3% in the intervention group; 15/150, 10.0% in the control group), 11.7% (35/300; 18/150, 12.0% in the intervention group; 17/150, 11.3% in the control group), and 13.3% (40/300; 17/150, 11.3% in the intervention group; 23/150, 15.3% in the control group) at 3-, 6-, and 9-month follow-ups, respectively. The average completion rate of the 3-month Run4Love program among people in the intervention group was 50.8% (33/65) [33].

### Results of LGCMs

**Figure 3:**
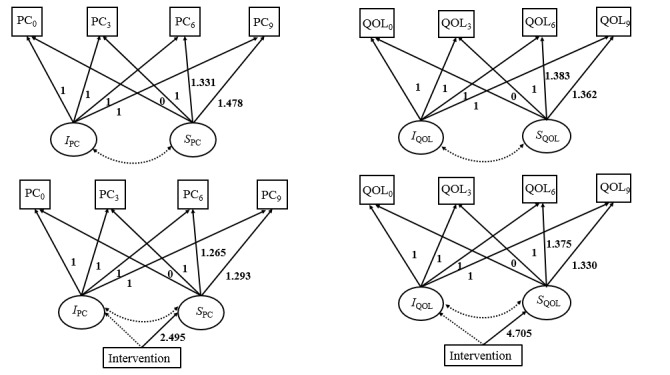
Path diagrams of the unconditional latent growth curve models (LGCMs) for quality of life (QOL) and positive coping and the conditional LGCMs with intervention groups as a covariate. Observed variables are denoted by boxes. Latent variables are denoted by ovals. Unidirectional arrows indicate the effects of 1 variable on the other. Bidirectional arrows indicate the correlations. The nonsignificant paths are shown as dotted lines. Intervention is either the Run4Love intervention group or the waitlist control group. I: intercept; PC: positive coping; S: slope; 0: baseline; 3: 3-month follow-up; 6: 6-month follow-up; 9: 9-month follow-up.

The results of the unconditional LGCMs indicated that both QOL and positive coping improved across the course of the study, and the largest improvement occurred at the 3-month follow-up. The path diagrams of these 2 unconditional LGCMs are presented in Figure 3. The unconditional LGCM for QOL had a good model fit: *χ*^2^_3_=2.7 (*P*=.26), CFI=0.999, TLI=0.996, SRMR=0.044, and RMSEA=0.034 (Figure 3). The mean intercept was 77.032 (*P*<.001, SE 0.527), indicating a mean QOL score of 77.032 at baseline. The mean slope was 2.241 (*P*<.001, SE 0.503), and the factor loadings on the slope were 0, 1, 1.383, and 1.362 at baseline and 3, 6, and 9 months, respectively. These results reflected a nonlinear pattern of improvement in QOL: a rapid increase in QOL scores (2.241 points) occurred at 3 months, and then the magnitude of improvement flattened out. The variances of the intercept and the slope of QOL were 92.509 (*P*=.001) and 54.013 (*P*=.04), respectively, which indicated significant individual differences in the initial levels and rates of change in QOL over time. The covariance between the intercept and the slope of QOL was −16.029 (*P*=.48), implying that the rate of change in QOL was not associated with its initial levels. The unconditional LGCM for positive coping also had a good model fit: *χ*^2^_3_=1.6 (*P*=.66), CFI=1.000, TLI=1.000, SRMR=0.025, and RMSEA<0.001 (Figure 3). The mean positive coping score was 18.373 (*P*<.001, SE=0.340) at baseline, and the mean slope was 0.699 (*P*=.04, SE=0.336). Similarly, the results of the slope factor also reflected a nonlinear pattern of improvement in positive coping: a rapid increase in positive coping scores (0.699 points) occurred at 3 months, and then the magnitude of improvement flattened out. The variances of the intercept and the slope in positive coping were 21.571 (*P*=.01) and 10.194 (*P*=.21), respectively, indicating significant individual differences in the initial levels of positive coping but not in its rates of change. The rate of change in positive coping was also unrelated to its initial levels, with a covariance between the intercept and the slope of −2.664 (*P*=.70).

The results of the conditional LGCMs indicated that the mHealth intervention had significantly positive effects on both QOL and positive coping across the course of the study. The path diagrams of these 2 conditional LGCMs are also presented in Figure 3. The conditional model for QOL fitted the data well: *χ*^2^_5_=3.3 (*P*=.50), CFI=1.000, TLI=1.000, SRMR=0.041, and RMSEA<0.001 (Figure 3). The intervention had a significant impact on the slope factor (*b*=4.705, SE=0.911, *P*<.001), indicating an improvement in QOL in the intervention group compared with the control group over time. Similarly, the conditional model for positive coping fitted the data well: *χ*^2^_5_=5.7 (*P*=.34), CFI=0.997, TLI=0.995, SRMR=0.030, and RMSEA=0.022 (Figure 3). There was a higher rate of change in positive coping in the intervention group (*b*=2.495, SE=0.826, *P*=.003), indicating a larger improvement in positive coping in the intervention group.

**Figure 4:**
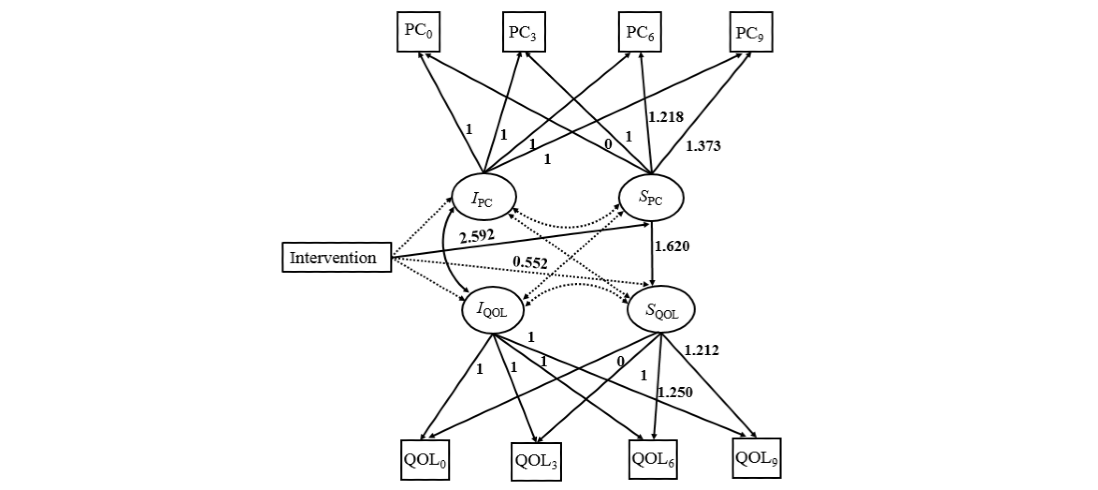
Path diagram of a parallel process latent growth curve model for quality of life (QOL) and positive coping with intervention groups as a covariate. Observed variables are denoted by boxes. Latent variables are denoted by ovals. Unidirectional arrows indicate the effects of 1 variable on the other. Bidirectional arrows indicate the correlations. The nonsignificant paths are shown as dotted lines. Intervention is either the Run4Love intervention group or the waitlist control group. I: intercept; PC: positive coping; S: slope; 0: baseline; 3: 3-month follow-up; 6: 6-month follow-up; 9: 9-month follow-up.

The results of the parallel process LGCM indicated that the mHealth intervention was effective in improving QOL via the mediation effect of positive coping. The path diagram of the parallel process LGCM is presented in Figure 4, and the estimates of the main coefficients are shown in Table 4. The results showed a good model fit for the parallel process LGCM: *χ*^2^_24_=64.1 (*P*<.001), CFI=0.971, TLI=0.957, SRMR=0.065, and RMSEA=0.075 (Figure 4). The intervention had a significantly positive impact on the slope of positive coping (*b*=2.592, *P*<.001), which in turn had a significantly positive impact on the slope of QOL (*b*=1.620, SE=0.321, *P*<.001). The indirect effect of the intervention on the slope of QOL via the slope of positive coping was significant (*b*=2.592×1.620=4.198, *P*=.006). These results indicated a mediation effect of positive coping on patients’ QOL, where the exposure to the mHealth intervention significantly improved participants’ positive coping over time, which in turn led to a positive change in QOL. The intervention no longer had direct effects on the slope of QOL (*b*=0.552, *P*=.69), indicating that the effects of the intervention on QOL might be explained by the mediation effect of positive coping. There were no significant differences as a function of intervention in the initial status of either repeated measures, indicating no group differences in the starting point for either positive coping or QOL. The covariance between the 2 intercepts was 21.571 (*P*=.01), indicating that initial levels of positive coping were positively related to initial status with respect to QOL. In other words, participants who reported higher levels of positive coping at baseline tended to report higher levels of QOL at the same time. However, neither the covariance between the intercept of positive coping and the slope of QOL (2.941, *P*=.26) nor the covariance between the intercept of QOL and the slope of positive coping (−3.900, *P*=.16) was statistically significant. These results indicated that the initial level of positive coping was not significantly associated with the rate of change in QOL, nor was the initial level of QOL and the rate of change in positive coping.

**Table 4:** Estimates of the coefficients in the parallel process latent growth curve model for quality of life and positive coping (n=300).

Coefficient				Estimate		95% CI		Standardized estimate		SE		*P* value
Intervention→QOL^a^				0.552		−2.154 to 3.258		0.036		1.381		.69
Intervention→positive coping				2.592		1.124 to 4.060		0.398		0.749		.001
Positive coping→QOL				1.620		0.997 to 2.243		0.692		0.318		<.001
**Total effect**				4.750		2.766 to 6.734		0.311		1.012		<.001
	**Direct effect**											
		Intervention→QOL	0.552		−2.154 to 3.258		0.036		1.381		.69	
	**Indirect effect**											
		Intervention→positive coping→QOL	4.198		1.189 to 7.207		0.275		1.535		.006	

^a^QOL: quality of life.

## Discussion

### Principal Findings

To the best of our knowledge, our secondary analysis of data from the Run4Love study is among the first efforts to examine the mediating role of positive coping in patients’ QOL in an mHealth-based RCT among people living with HIV. The results of LGCMs demonstrated that the Run4Love trial significantly improved positive coping among people living with HIV over 9 months, and the enhancement of positive coping led to significant improvement in QOL across the study. There was full mediation between positive coping and QOL in the Run4Love trial. Thus, our study revealed one of the potential mechanisms of improvement in QOL in mHealth-based CBSM interventions. To better design and implement effective interventions for people living with HIV, more studies are needed to investigate and identify the processes and/or mechanisms by which interventions lead to significant improvements in health outcomes, especially in emerging mHealth interventions [27].

Although several cross-sectional studies and 1 cohort study have examined the mediating role of positive coping on QOL [19-21], such designs are not sufficient for a robust conclusion because their findings are based on passive observational data. An experimental design in which the proper intervention is used to improve an individual’s positive coping and QOL would greatly reduce confounding effects and enhance the strength of causal inferences. However, few RCTs have explored this relationship, especially in mHealth interventions. Only 1 face-to-face RCT showed that improvement in positive coping significantly mediated the effects of psychosocial intervention on QOL using pre- and postintervention assessments [25]. A comprehensive literature review suggested that mediation analysis could only be properly examined in well-designed RCTs with repeated measurements and sufficient power [27]. Thus, our study is well suited to investigate such a relationship and has confirmed the mediating role of positive coping on QOL, which may expand the literature on mechanisms of mHealth-based interventions.

In addition to the RCT design, the time point repeated measures and corresponding methodology of LGCM used in this study allow more conclusive findings and more abundant information for the mediation analysis or other research. Unlike cross-sectional data or pre- and postintervention data adopted in the previous literature, LGCMs that use longitudinal data allow the trajectory estimation of changes in outcome measures over time [39,40]. For example, the results of the LGCMs in our study suggested that both positive coping and QOL had increasing and nonlinear trajectories with a slower rate of increase over time, and there were statistically significant individual differences in the initial levels and rates of change of positive coping and QOL over time. In addition, the parallel process LGCM allowed for the examination of intercept-intercept, intercept-slope, and slope-slope relationships of the trajectories, which might extend our understanding of the relationships between the factors investigated [46]. In our study, we found a significant slope-slope relationship between positive coping and QOL, which indicated a mediation effect of positive coping on QOL in the mHealth intervention. Therefore, the LGCMs could not only estimate the growth curve of each outcome measure over time but also simultaneously examine the mediation relationship between 2 outcomes (eg, positive coping and QOL) in an intervention by controlling for their growth trajectories [39].

Given the critical role of positive coping in improving QOL, it is important to develop positive coping skills in mHealth interventions to improve participants’ QOL. Previous psychosocial interventions for people living with HIV found that training in active cognitive and adaptive behavioral coping strategies was effective in improving positive coping [23,47,48]. In the Run4Love trial, we adapted the evidence-based CBSM intervention with important components of training in various types of coping skills, such as active cognitive coping (eg, mindfulness and problem- or emotion-oriented coping) and adaptive behavioral coping (eg, regular exercise) [28]. In addition, we adapted the CBSM courses originally designed for people living with HIV in the United States by removing some parts of the courses that were not suitable in the Chinese context, such as religion-related materials. Therefore, we had 9 sessions out of the original 10 sessions of the CBSM courses. Furthermore, as Chinese people living with HIV visit the hospital for antiretroviral drugs and regular clinical follow-ups every 3 months, we matched the patients’ hospital visits and adapted the length of the intervention to 3 months by adding 3 review sessions and having weekly delivery of the total 12 sessions of the CBSM courses [28]. In addition, our CBSM intervention delivered in the mHealth platform allowed and encouraged participants’ repeat visits to the training items, which might result in enhanced and sustained intervention effects over 9 months by repeatedly accessing the CBSM courses and practicing the coping skills, thus producing improved and sustained effects on patient outcomes such as QOL. To our knowledge, there is limited evidence about the long-term effects of mHealth interventions; few studies have reported intervention effects for over 6 months [49,50]. Few studies have been conducted on the potential mechanisms of the persistent intervention effects in mHealth interventions. The investigation in this study may provide important information and evidence on intervention mechanisms for long-term sustained effects in mHealth interventions.

### Limitations

This study had several limitations. First, the self-reported data on positive coping and QOL in our study might have resulted in recall and social desirability biases. More objective measures, such as biomarkers, could be incorporated in future studies. Second, although participants were recruited from a large hospital for HIV treatment in Guangzhou with over 10,000 patients who were HIV seropositive, the sample was mostly from an urban setting and was predominantly male, particularly young men who have sex with men. Therefore, the generalizability of our findings should be treated with caution. Third, as all the 6 dimensions of QOL were improved in the intervention group at 3, 6, and 9 months, and the improvement was not limited to the mental health dimension, it is possible that the improvement in QOL was also related to other factors besides positive coping, such as reduced depressive symptoms, stress, and/or HIV-related stigma and increased social support [51-53]. Although it is beyond the scope of this study to incorporate other factors, one of our previous studies found mediating roles of perceived stress and depressive symptoms for suicide reduction [53], whereas another study found positive coping and HIV-related stigma as intervention mediators on depressive symptoms [52]. Therefore, other potential mediators should be further explored to clarify the mechanisms of intervention effects and processes of how mHealth interventions improve health outcomes for better designing and implementing effective mHealth interventions.

### Conclusions

In conclusion, this study found a full mediation effect of positive coping on QOL among people living with HIV in an mHealth intervention using 4 time point repeated measures and LGCMs. Future research and policies aimed at QOL improvement among people living with HIV should be designed with specific features that enhance the use of positive coping strategies.

## Abbreviations

CBSM: cognitive-behavioral stress management
CFI: confirmatory fit index
LGCM: latent growth curve model
mHealth: mobile health
QOL: quality of life
RCT: randomized controlled trial
RMSEA: root mean square error of approximation
SRMR: standardized root mean square residual
TLI: Tucker-Lewis index
WHOQOL-HIV BREF: World Health Organization Quality of Life HIV short version
